# Paraneoplastic dermatomyositis associated with metastatic leiomyosarcoma of unknown primary

**DOI:** 10.1186/s13569-020-00140-w

**Published:** 2020-08-25

**Authors:** Eve Merry, Alannah Smrke, Kapil Halai, Gulam Patel, Khin Thway, Robin L. Jones, Charlotte Benson

**Affiliations:** 1 Sarcoma Unit, The Royal Marsden Hospital NHS Foundation Trust, 203 Fulham Road, London, SW3 6JJ UK; 2 Rheumatology Department, Ashford and St Peter’s NHS Hospitals NHS Foundation Trust, London, TW15 3AA UK; 3 The Institute of Cancer Research, 237 Fulham Road, London, SW3 6JB UK

**Keywords:** Dermatomyositis, Leiomyosarcoma, Soft tissue sarcoma, Chemotherapy, TIF1γ antibody, Paraneoplastic

## Abstract

**Background:**

Sarcomas are rare and heterogeneous tumours of mesenchymal origin, with over 100 histological subtypes. Paraneoplastic dermatomyositis has rarely been described in sarcoma. This is the first documented case of paraneoplastic dermatomyositis in a patient with metastatic leiomyosarcoma.

**Case presentation:**

A 43-year-old female diagnosed with metastatic leiomyosarcoma of unknown primary presented with a mild rash in sun-exposed areas of her face and upper chest, with no other neuromuscular symptoms. This rash resolved with systemic treatment with doxorubicin for metastatic leiomyosarcoma. Imaging assessment confirmed overall stable disease after chemotherapy completion. She presented acutely 2 months later with new onset rash in a shawl-like distribution, periorbital oedema and proximal muscle weakness. Based on the characteristic cutaneous signs and symmetrical proximal muscle weakness, abnormal electromyography and raised skeletal muscle enzymes with a positive anti-transcription intermediary factor-1 gamma antibody result, a diagnosis of paraneoplastic dermatomyositis was made. Re-evaluation of her metastatic leiomyosarcoma revealed disease progression. Second-line chemotherapy was commenced once the dermatomyositis was controlled on steroid therapy. Systemic anti-cancer therapy was again associated with mild improvement in dermatomyositis symptoms.

**Discussion:**

Paraneoplastic dermatomyositis heralded disease progression after first-line chemotherapy; however, in hindsight, subtle cutaneous features were present at sarcoma diagnosis. The temporal relationship between paraneoplastic dermatomyositis and metastatic leiomyosarcoma is key in this case, as fluctuations in dermatomyositis severity correlated with growth of metastatic disease. Understanding this relationship may provide clues for tumour progression and prompt timely initiation of anti-cancer therapy. It is important to recognise that in addition to the more common cancers associated with paraneoplastic dermatomyositis, it can also occur in rarer tumours such as leiomyosarcoma.

## Introduction

Dermatomyositis is a form of autoimmune inflammatory myopathy, with characteristic cutaneous features and myositis-related weakness [1]. Stertz first described the association between dermatomyositis and visceral malignancy in 1916, in a patient with gastric carcinoma [2]. The underlying mechanism of paraneoplastic dermatomyositis remains incompletely understood. A leading hypothesis is that there is cross-reactivity with tumour-directed autoantibodies attacking similar autoantigens within muscle and skin cells [3]. Paraneoplastic dermatomyositis has been most commonly observed in ovarian, lung, pancreatic, stomach and colorectal cancers [4, 5]. Sarcomas are rare and heterogeneous cancers with over 100 different histologic subtypes [6] and are uncommonly associated with paraneoplastic syndromes. A small number of cases of paraneoplastic dermatomyositis have been described in chondrosarcoma [7–10] and one case in a soft tissue neoplasm characterised only as ‘low-grade mesenchymal neoplasm’ [11]. To our knowledge, the association between paraneoplastic dermatomyositis and leiomyosarcoma has not been reported. Here, we report a case of a patient presenting with paraneoplastic dermatomyositis associated with metastatic leiomyosarcoma of unknown primary.

## Case report

A 43-year-old, previously healthy Caucasian female presented to her general practitioner with a few-months history of left knee pain. She had also noticed a left-sided supraclavicular fossa (SCF) mass. She had no significant medical history, never smoked, with moderate alcohol intake. A maternal grandmother had endometrial cancer aged 65 years.

An ultrasound-guided core biopsy of the suspicious SCF mass was urgently arranged. Review by an expert soft tissue pathologist revealed grade 2 leiomyosarcoma, with immunohistochemistry positive for desmin, smooth muscle actin and h-caldesmon. CT and PET-CT imaging showed extensive metastatic disease, with no clear primary site. There were widespread cutaneous, subcutaneous, soft tissue, lung, liver, right adrenal and peritoneal metastases. A lytic lesion involving the left femoral head was identified as at risk for pathological fracture and a likely cause of the patient’s longstanding left knee pain. She initially underwent urgent prophylactic stabilisation of the left femur, followed by radiotherapy.

She commenced first-line palliative doxorubicin chemotherapy. The patient was noted to have a mild erythematous maculopapular rash on her face and upper chest in sun-exposed areas at her pre-treatment consultation. This developed several days following femoral surgery and persisted for a few weeks. No drug, environmental or infective triggers were identified. The rash was treated with topical hydrocortisone 1% and emollient cream (diprobase), and resolved during cycle one of doxorubicin with a sustained remission throughout chemotherapy. She did not report any muscle weakness or pain during this time. Six cycles of doxorubicin were well tolerated, with one episode of febrile neutropenia requiring a 25% dose reduction. Imaging assessment at the end of chemotherapy showed overall stable metastatic disease by RECIST 1.1 criteria [12], with a minor reduction in some metastatic deposits noted mid-treatment.

**Fig. 1:**
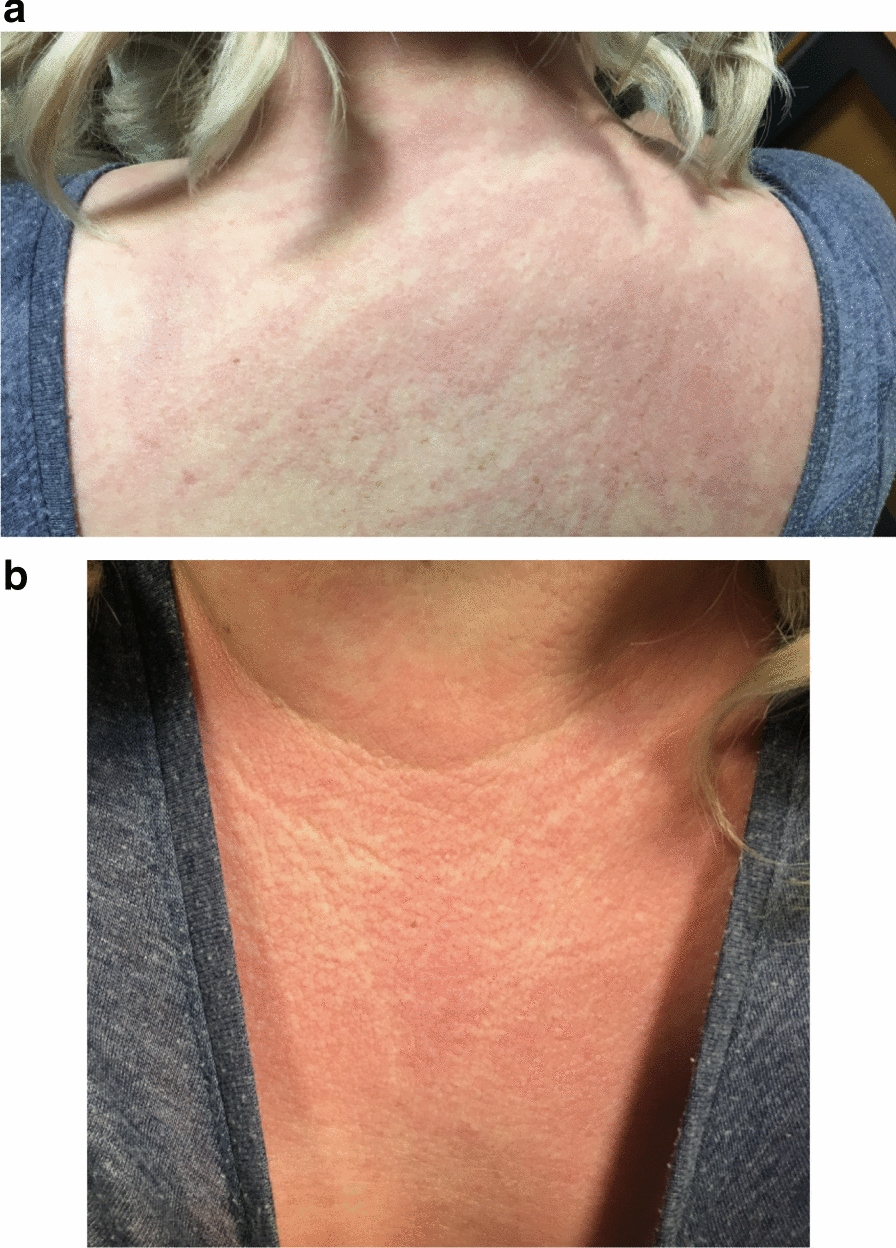
Clinical features of dermatomyositis with shawl-like rash affecting neck, chest and back. **a** Flagellate erythema upper back. **b** ‘V’ neck distribution of erythema

**Fig. 2:**
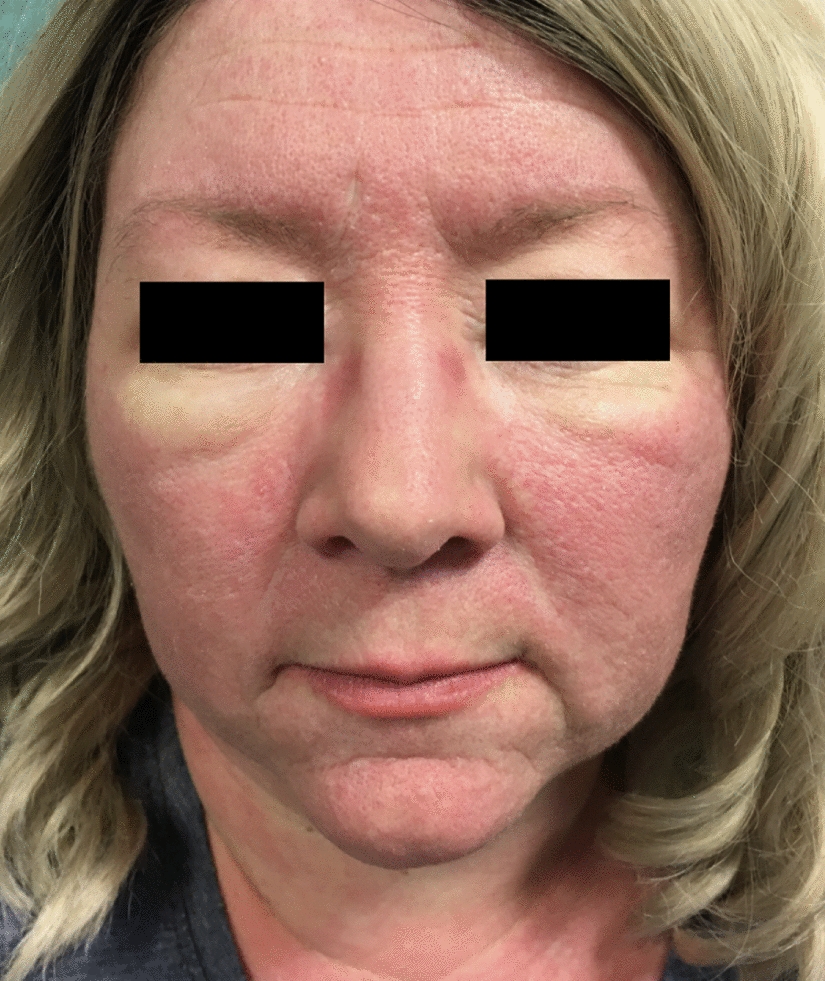
Clinical features of dermatomyositis including facial heliotrope rash and periorbital oedema

Two months later, the patient presented acutely unwell to her local hospital with a new-onset rash in a shawl-like distribution (upper chest, neck and arms), with flagellate erythema (Fig. 1) and associated periorbital oedema (Fig. 2) and proximal limb weakness. The patient denied breathing or swallowing difficulty. On examination periungual erythema and Gottron’s papules were noted, without visible dilated capillary nailfold loops. There was reduced proximal power in both upper and lower limb (3–4/5), but no neck or truncal weakness. Vital signs found she was tachycardic and febrile. Routine blood tests on admission showed c-reactive protein < 4 and mildly raised white cell count 11.4 (normal range 4–11 × 10^9^L). Alanine aminotransferase (ALT) was mildly raised at 61 (normal 10–49 U/L) with unremarkable renal function. Blood cultures grew Staphylococcus hominis and she was treated with intravenous antibiotics and appropriate medical management.

Rheumatology and dermatology review led to the diagnosis of dermatomyositis, likely paraneoplastic, in the context of known metastatic leiomyosarcoma. Investigation supporting this included; raised creatinine kinase (CK) and erythrocyte sediment rate at 695 (normal range 25–200 U/L) and 32 (normal range 0–27 mm/h) respectively. Autoantibody screen negative for ENA, dsDNA, anti-CCP, with normal complement levels and weakly positive ANA (1:160). Myositis antibody panel was positive for transcription intermediary factor-1 gamma (TIF1γ) antibodies alone, whilst anti-Jo1 and anti-Mi2 antibody negative. In addition to intramuscular metastatic disease, MRI femur showed mild increased signal within the muscles compatible with myositis. An echocardiogram confirmed normal cardiac function. Electromyography (EMG) found myopathic changes of moderate degree in lower and upper limbs, worse in the lower limbs with proximal predominance. The findings were in keeping with a diagnosis of proximal myopathy. Right chest punch biopsy histology was consistent with dermatomyositis. Muscle biopsy was not performed, given the potential risk of metastatic disease seeding.

Treatment was commenced with a weaning course of prednisolone (initial dose 40 mg daily) and topical clobetasol 0.05% with clinical improvement in both weakness and rash over several months. Diagnosis of paraneoplastic dermatomyositis triggered re-evaluation of the patient’s leiomyosarcoma with CT imaging, which showed multifocal progression. Second-line chemotherapy with trabectedin was commenced following recovery from the acute admission and once the dermatomyositis was controlled with steroid therapy. Systemic anti-cancer therapy was again associated with mild improvement in dermatomyositis symptoms.

## Discussion

Paraneoplastic dermatomyositis was first described in 1916 and represents approximately 30% of dermatomyositis cases [2, 4].Population-based studies have supported an increased risk of cancer in dermatomyositis patients, with standardised incidence ratios for cancer between 3.0 and 7.7 [4, 5, 13, 14]. Strongest associations have been seen with ovarian, lung, pancreatic, stomach and colorectal cancers [4, 5], and tumours with high prevalence in specific populations, namely nasopharyngeal carcinoma in a southeast Asian population [13]. Paraneoplastic dermatomyositis has been seen with chondrosarcoma (4 cases) [7–10] and an unspecified soft tissue sarcoma (1 case) [11]. Like other paraneoplastic phenomena, the pathophysiology of paraneoplastic dermatomyositis is thought to relate to autoimmune cross-reactivity between similar autoantigens within the cancer tissue and normal tissue in muscle and skin.^3^

**Fig. 3:**
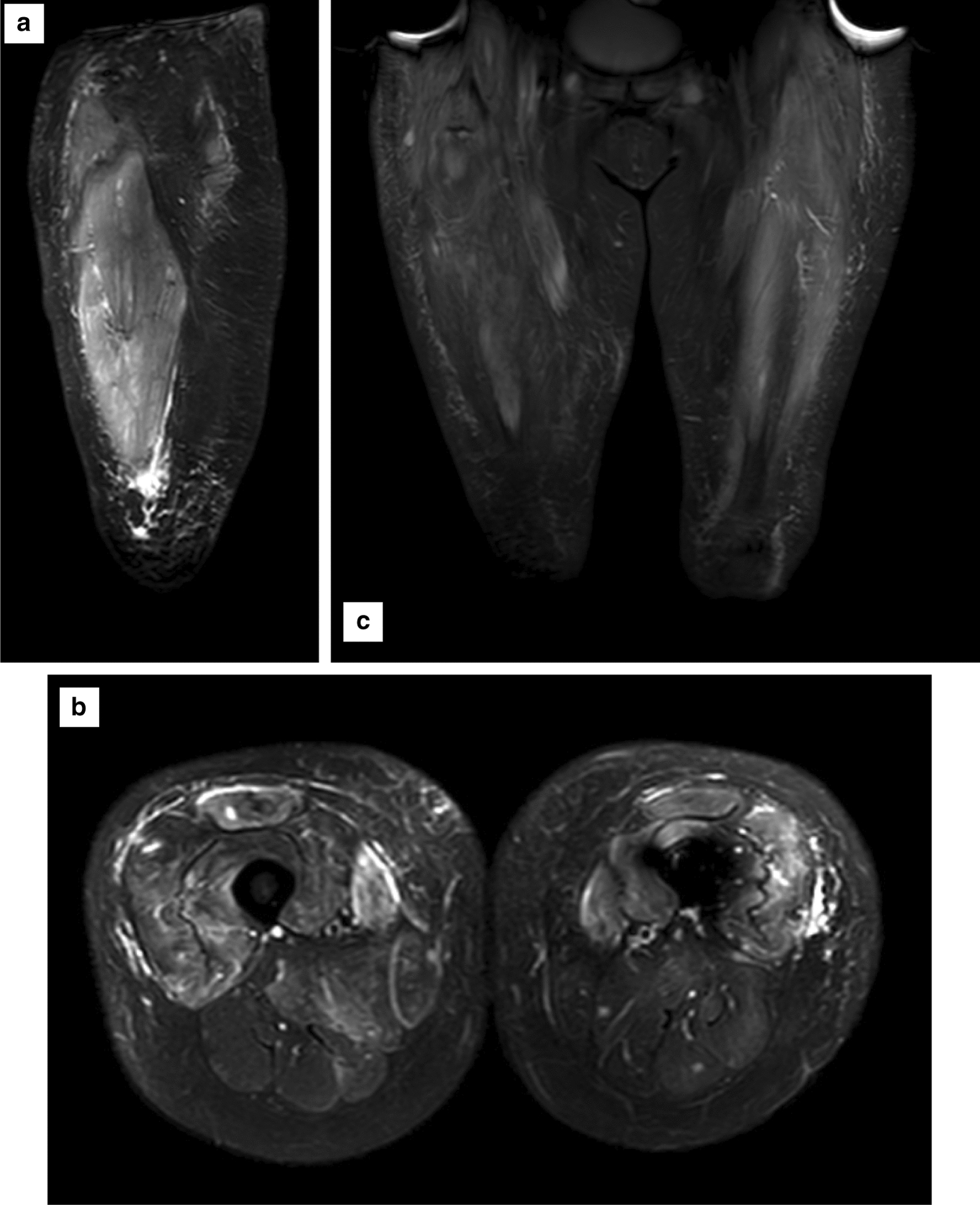
MRI signal change of the musculature of the lower limbs on (**a**) left sagittal (**b**) axial (**c**) coronal inversion recovery sequence in keeping with myositis

The diagnosis of paraneoplastic dermatomyositis is supported by both the clinical and serological features found in this case. Bohan and Peter proposed five diagnostic criteria (Table 1) for dermatomyositis in 1975 [15, 16]. Our patient displayed four out of five of these criteria with the absent criterion being a positive muscle biopsy, which was performed due to high risk for metastatic spread. MRI did show signal changes consistent with myositis (Fig. 3). MRI imaging has been used for diagnosis and assessment of treatment response for inflammatory myositis [17]. The results of MRI imaging coupled with the patient’s clinical presentation fit diagnostic criteria without the need for biopsy.

**Table 1:** Bohan and Peter’s proposed diagnostic criteria for dermatomyositis

Criterion
I Symmetrical proximal muscle weakness ± dysphagia or respiratory muscle involvement
II Increase of skeletal muscle enzymes; creatine kinase, aspartate aminotransferase, alanine aminotransferase and lactate dehydrogenase
III Abnormal EMG characteristic of myopathy
IV Abnormal muscle biopsy
V Typical cutaneous features (e.g. Gottron’s sign, heliotrope rash with periorbital oedema)
*Definite* diagnosis: Criterion V and at least 3 of criteria I–IV *Probable* diagnosis: Criterion V and 2 of criteria I–IV *Possible* diagnosis: Criterion V and 1 of criteria I–IV

The Bohan and Peter diagnostic criteria requires presence of characteristic skin manifestations alongside three out of the four remaining criteria, symmetrical proximal muscle weakness, increase of skeletal muscle enzymes (namely ALT and CK), abnormal EMG and typical skin rash, for a definite diagnosis as seen in this case [15, 16, 18, 19]. In addition, this patient also meets the definite criteria of the Paraneoplastic Neurological Syndrome Euronetwork for paraneoplastic neurological syndrome, with dermatomyositis as the classical syndrome and a cancer developing within 5 years of dermatomyositis diagnosis [20]. While the Bohan and Peter classification remains widely used for the diagnosis of dermatomyositis [21], updated diagnostic criteria combining clinical features and serological testing, including myositis-specific and myositis-associated antibodies have been proposed [21, 22]. The goal of updates to the classification is to ensure robust, reliable criteria for clinical trial enrolment and understand the distinct clinical behaviour of different inflammatory myopathies. However, recent EULAR/ACR consensus criteria lack incorporation of MRI findings and non anti-Jo1 antibodies [23], leaving room for improvement of this contemporary diagnostic criteria.

In our case, serological results support paraneoplastic dermatomyositis, with an isolated TIF1γ antibody positive test. The presence of the TIF1γ antibody indicates increased risk of cancer-associated dermatomyositis with high specificity and moderate sensitivity [24, 25]. A meta-analysis assessed the clinical value of a positive TIF1γ antibody test to predict cancer-associated dermatomyositis, and concluded a positive predictive value of 58%, and a negative predictive value of 95% [26]. TIF1γ antibody-positive dermatomyositis and cancer has been more commonly seen in older patients (age over 39 years). It has a different phenotypic expression in children or young adults, with skin ulceration and chronic disease, without a cancer association [27, 28]. The only other reported association between TIF1γ antibody-positive dermatomyositis and sarcoma has been described in a Caucasian woman of similar age with chondrosarcoma of the humerus [10]. Importantly, the absence of other myositis antibodies in this patient, including anti-Jo1 and anti-Mi2, is also supportive of paraneoplastic dermatomyositis [25]. In a cross-sectional study of UK Caucasian adults with dermatomyositis, patients without myositis-specific autoantibodies, like anti-Jo1 and anti-Mi2, had a significantly increased risk of cancer-associated dermatomyositis [25]. They concluded that a negative routine myositis antibody panel is highly sensitive for cancer associated dermatomyositis.

The diagnosis of cancer has been reported to occur before, at the same time or after dermatomyositis onset [14, 29]. Most cancers are detected within the first year of diagnosis of dermatomyositis and the risk remains high for up to 5 years [25]. A cohort study focusing specifically on TIF1γ antibody-positive dermatomyositis patients found that malignancy occurred between 3 years prior and 2.5 years after dermatomyositis diagnosis [29]. In this case the diagnosis of metastatic leiomyosarcoma of unknown primary preceded the TIF1γ-positive dermatomyositis diagnosis by several months. Resolution of dermatomyositis has been variably associated with curative-intent surgical excision and optimal immunosuppressive treatment [11]. Symptoms have been reported to resolve in a case of resected soft tissue sarcoma [11]. In contrast, surgical excision of a humeral chondrosarcoma did not lead to complete resolution of dermatomyositis but control of the cutaneous features improved [10]. In a case of paraneoplastic dermatomyositis related to a cavernous sinus chondrosarcoma, complete surgical excision was not possible and the skin lesions persisted despite appropriate steroid and immunosuppressive therapy [30]. These limited cases suggest that even with optimal medical treatment for dermatomyositis, in patients with unresectable or metastatic disease, dermatomyositis does not fully resolve.

The severity of the skin lesions appeared to wax and wane, with the metastatic disease suggesting a temporal relationship between cutaneous features and leiomyosarcoma disease status. In patients with paraneoplastic dermatomyositis and advanced cancer, similar temporal relationships have been described [31, 32]. The development of erythema in the ‘V’ neck distribution, in hindsight, may have been the first early sign of dermatomyositis and mild progression of metastatic disease. The subsequent severe, acute presentation with severe dermatomyositis, complicated by septicaemia, was a dramatic, and alarming red flag for cancer progression, as confirmed on imaging. Severe dermatomyositis complicated by septicaemia was also seen in uterine carcinosarcoma and highlights the potential seriousness of this paraneoplastic condition if untreated [33]. Now that this pattern has been identified, the clinical severity of dermatomyositis may predict response to anti-cancer therapy for metastatic leiomyosarcoma. Unfortunately, given the metastatic nature of the leiomyosarcoma, treatment intent is palliative and it is unlikely that the dermatomyositis will resolve completely. However, good control has been achieved with steroid therapy without the need for alternative immunosuppressive therapy. Understanding the relationship between the dual pathologies alongside multidisciplinary management between rheumatology and oncology teams is key to providing optimal treatment for paraneoplastic dermatomyositis.

## Conclusion

This is, to our knowledge, the first description of paraneoplastic dermatomyositis associated with leiomyosarcoma. Given leiomyosarcoma is a cancer of smooth muscle, it seems feasible that like other tumour types, the tumour cells may harbour autoantigens that mimic antigen within regenerating normal muscle allowing for autoimmune cross-reactivity and resultant myositis [3]. The temporal relationship between dermatomyositis and cancer diagnosis varies. Cancer risk is significantly elevated in patients with dermatomyositis, with the risk for cancer diagnosis highest in the first year [13]. Given the poor prognosis associated with metastatic leiomyosarcoma, and soft tissue sarcomas in general, it is important for physicians to be aware in the work-up of dermatomyositis, that there may be an underlying rare cancer. Early diagnosis of a sarcoma may provide the opportunity for curative surgical resection, and possible resolution of the dermatomyositis that heralded their disease. This case emphasises that multi-disciplinary management is key for patients with paraneoplastic dermatomyositis.

## Data Availability

Not applicable.

## Abbreviations

SCF: Supraclavicular fossa
US: Ultrasound
CT: Computerised Tomography
PET: Positron emission tomography
FDG: Fluorodeoxyglucose
RECIST: Response Evaluation Criteria in Solid Tumours
CRP: C-reactive protein
ALT: Alanine Aminotransferase
CK: Creatine kinase
ESR: Erythrocyte Sedimentation Rate
ENA: Extractable nuclear antigen
dsDNA: Double-stranded Deoxyribonucleic acid
CCP: Cyclic citrullinated peptides
ANA: Antinuclear antibodies
TIF1γ: Transcription intermediary factor-1 gamma
MRI: Magnetic resonance imaging
EMG: Electromyography
UK: United Kingdom
EULAR/ACR: European League Against Rheumatism/American College of Rheumatology

## References

[1] Dalakas MC, Hohlfeld R (2003). Polymyositis and dermatomyositis. Lancet.

[2] G S. Polymyositis Berl Klin Wochenschr. 1916;53:489.

[3] Casciola-Rosen L, Nagaraju K, Plotz P, Wang K, Levine S, Gabrielson E (2005). Enhanced autoantigen expression in regenerating muscle cells in idiopathic inflammatory myopathy. J Exp Med.

[4] Hill CL, Zhang Y, Sigurgeirsson B, Pukkala E, Mellemkjaer L, Airio A (2001). Frequency of specific cancer types in dermatomyositis and polymyositis: a population-based study. Lancet.

[5] Stockton D, Doherty V, Brewster D (2001). Risk of cancer in patients with dermatomyositis or polymyositis, and follow-up implications: a Scottish population-based cohort study. Br J Cancer.

[6] Brennan MF, Antonescu CR, Maki RG. Management of soft tissue sarcoma: Springer; 2013.

[7] Baker MC, Smith GP, Miloslavsky EM (2019). Dermatomyositis associated with a skull base chondrosarcoma. J Clin Rheumatol.

[8] Patel SJ, Sanjana NE, Kishton RJ, Eidizadeh A, Vodnala SK, Cam M (2017). Identification of essential genes for cancer immunotherapy. Nature.

[9] Mol MT, Stalenhoef A, Boerbooms AT (1986). Chondrosarcoma coexistent with dermatomyositis. J Am College Rheumatol.

[10] Dziwis J, Agnihothri R, Lieberman A, Richardson CT (2019). A unique case of dermatomyositis associated with anti-TIF1γ antibody and chondrosarcoma. JAAD case Rep.

[11] Ali M, Sendur N, Aksoy S, Yaman S, Arik Z, Kilinç L (2012). Dermatomyositis complicated with a soft tissue sarcoma. Rheumatol Int.

[12] Eisenhauer EA, Therasse P, Bogaerts J, Schwartz LH, Sargent D, Ford R (2009). New response evaluation criteria in solid tumours: revised RECIST guideline. Eur J Cancer.

[13] Chen Y-J, Wu C-Y, Huang Y-L, Wang C-B, Shen J-L, Chang Y-T (2010). Cancer risks of dermatomyositis and polymyositis: a nationwide cohort study in Taiwan. Arthritis Res Ther.

[14] Sigurgeirsson B, Lindelöf B, Edhag O, Allander E (1992). Risk of cancer in patients with dermatomyositis or polymyositis. N Engl J Med.

[15] Bohan A, Peter J (1975). Medical progress I Polymyositis and dermatomyositis. N Engl J Med.

[16] Bap JB (1975). Polymyositis and dermatomyositis. N Engl J Med.

[17] May DA, Disler DG, Jones EA, Balkissoon AA, Manaster B (2000). Abnormal signal intensity in skeletal muscle at MR imaging: patterns, pearls, and pitfalls. Radiographics.

[18] Iaccarino L, Ghirardello A, Bettio S, Zen M, Gatto M, Punzi L (2014). The clinical features, diagnosis and classification of dermatomyositis. J Autoimmun.

[19] Muro Y, Sugiura K, Akiyama M (2016). Cutaneous manifestations in dermatomyositis: key clinical and serological features—a comprehensive review. Clin Rev Allergy Immunol.

[20] Graus F, Delattre J, Antoine J, Dalmau J, Giometto B, Grisold W (2004). Recommended diagnostic criteria for paraneoplastic neurological syndromes. J Neurol Neurosurg Psychiatry.

[21] Leclair V, Lundberg IE (2018). New Myositis Classification Criteria—What We Have Learned Since Bohan and Peter. Curr Rheumatol Rep.

[22] Benveniste O, Stenzel W, Allenbach Y (2016). Advances in serological diagnostics of inflammatory myopathies. Curr Opin Neurol.

[23] Lundberg IE, Tjärnlund A, Bottai M, Werth VP, Pilkington C, de Visser M (2017). EULAR/ACR classification criteria for adult and juvenile idiopathic inflammatory myopathies and their major subgroups. Arthritis Rheumatol.

[24] Lu X, Yang H, Shu X, Chen F, Zhang Y, Zhang S (2014). Factors predicting malignancy in patients with polymyositis and dermatomyostis: a systematic review and meta-analysis. PLoS ONE.

[25] Chinoy H, Fertig N, Oddis CV, Ollier WE, Cooper RG (2007). The diagnostic utility of myositis autoantibody testing for predicting the risk of cancer-associated myositis. Ann Rheum Dis.

[26] Trallero-Araguás E, Rodrigo-Pendás JÁ, Selva-O’Callaghan A, Martínez-Gómez X, Bosch X, Labrador-Horrillo M (2012). Usefulness of anti-p155 autoantibody for diagnosing cancer-associated dermatomyositis: a systematic review and meta-analysis. Arthritis Rheum.

[27] Waller R, Ahmad N (2019). TIF1-γ associated dermatomyositis. Rheumatol Adv Practice.

[28] Fiorentino D, Casciola-Rosen L (2012). Autoantibodies to transcription intermediary factor 1 in dermatomyositis shed insight into the cancer-myositis connection. Arthritis Rheum.

[29] Oldroyd A, Sergeant JC, New P, McHugh NJ, Betteridge Z, Lamb JA (2019). The temporal relationship between cancer and adult onset anti-transcriptional intermediary factor 1 antibody–positive dermatomyositis. Rheumatology.

[30] Patel MM, Stacy RC (2013). Paraneoplastic dermatomyositis related to a chondrosarcoma involving the cavernous sinus. J Neuroophthalmol.

[31] Nagano Y, Inoue Y, Shimura T, Fujikawa H, Okugawa Y, Hiro J (2015). Exacerbation of dermatomyositis with recurrence of rectal cancer: a case report. Case Rep Oncol.

[32] Ono K, Shimomura M, Toyota K, Kagimoto A, Tsukiyama N, Shishida M (2017). Successful resection of liver metastasis detected by exacerbation of skin symptom in a patient with dermatomyositis accompanied by rectal cancer: a case report and literature review. Surgical Case Rep.

[33] Chandiramani M, Joynson C, Panchal R, Symonds R, Brown L, Morgan B (2006). Dermatomyositis as a paraneoplastic syndrome in carcinosarcoma of uterine origin. Clin Oncol.

